# Annual Temperature Variation, Not Number of Predators, Predicts Variation in Foraging Group Size Among Pigeons Worldwide

**DOI:** 10.3390/biology14070757

**Published:** 2025-06-25

**Authors:** Guy Beauchamp

**Affiliations:** Independent Researcher, Montreal, QC, Canada; guygillesbeauchamp@gmail.com

**Keywords:** altitude, birds, group foraging, islands, latitude, predation risk

## Abstract

Many life history traits show predictable patterns at broad biogeographical scales, yet much less is known about how behavioural traits vary across such gradients. In this study, I investigated global variation in foraging group size within a large, cosmopolitan avian family—the pigeons—and assessed the roles of climate and predation in shaping these patterns. I compiled data from the literature for species occurring across a range of latitudes, altitudes, and habitats (islands v. continents) and analyzed the data within a phylogenetic framework that accounted for both biogeographical and ecological drivers of group size. Foraging group size increased with latitude among continental species but did not follow these trends on islands, where groups were generally smaller. Group size also increased at higher elevations. Among the climatic variables examined, annual temperature variation emerged as the strongest latitudinal predictor: greater temperature variability was associated with larger group sizes in continental species, but not in island species. The number of predators within a species’ breeding range showed no significant relationship with group size. These findings suggest that climatic variability, rather than number of predators, is a key driver of foraging group size in pigeons at the biogeographical scale.

## Simple Summary

While species diversity and traits linked to reproduction and survival are known to vary with latitude, altitude, and island residency, much less is known about how behavioural traits change across broad biogeographical gradients. Using data from the literature, I investigated global variation in foraging group size within a large avian family—the pigeons. Specifically, I examined how group size is related to climatic factors and predator richness. Group size tended to increase at higher latitudes and elevations and was generally smaller among island species. The number of predators within a species’ breeding range showed no significant association with group size. Among climatic variables, annual temperature variation was the strongest predictor of group size, with greater variation linked to larger groups. These findings suggest that, at a global scale, climatic factors—rather than predation—play a primary role in shaping variation in foraging group size in pigeons.

## 1. Introduction

Global variation in ecological conditions offers valuable insight into how animals adapt to diverse environments. Consequently, much research has focused on trait variation in animals across different latitudes, altitudes, and habitats. For example, species richness tends to decline at higher latitudes [1], where animals also tend to produce larger clutches [2] and have shorter lifespans [3]. Similarly, a reduction in species diversity is observed at higher elevations [4] and on islands [5]. Island species are further distinguished by slower life histories compared to their mainland counterparts [6]. However, in contrast to life history traits and patterns of biodiversity, behavioural traits have received relatively little attention in biogeographical research. Here, I examined how one such behavioural trait, namely, foraging in groups, varies as a function of latitude, altitude, and island living in one large, cosmopolitan avian family—the pigeons (Columbidae). Knowledge about variation in group size at the biogeographical scale is important to identify key environmental predictors and possible evolutionary advantages. For instance, a global analysis revealed that variability among years in precipitation was a strong predictor of cooperative breeding in birds, suggesting that sociality during the reproductive season might have evolved as a buffer against environmental uncertainty [7].

Animals form groups to perform various activities such as sleeping or foraging. While many species forage alone, other species can forage in very large groups numbering in the thousands [8]. Research suggests that living in groups has evolved to enhance foraging success and deter predation [9]. For instance, individuals in groups can feed more efficiently and safety in numbers through various mechanisms can reduce predation risk [10]. Research has thus far established how variation in ecological factors affects group size at the scale of the habitat [11,12,13]. Much less is known about variation in group size at broad biogeographical scales including latitude, altitude, and island living.

With respect to latitude, two theories can predict variation in foraging group size. If group foraging influences predation risk, larger groups are expected at lower latitudes in response to the occurrence of a larger number of predators as per the latitudinal gradient in biodiversity. The same argument was proposed to explain why nesting success in birds decreases at lower latitudes [14] and why more tropical species are more fearful [15], all of which are suggestive of generally more intense interactions between predators and prey at lower latitudes [16,17]. Alternatively, the more benign and less seasonal climate in tropical areas, as shown by reduced environmental harshness and increased thermal stability [18], could reduce the need to forage in groups. For instance, key resources in tropical areas may be available for longer periods, more abundant, and easier to locate [19]. Indeed, year-round territoriality in birds, which capitalizes on stable resources, is more common in the tropics [20] illustrating the reduced need to forage in groups in more benign and stable environments. By contrast to the predator richness argument, the climate stability hypothesis predicts an increase in group size at higher latitudes. The two theories make the same predictions with respect to altitude. This is because species richness typically decreases at higher altitudes mirroring the latitudinal gradient [4]. In addition, areas at higher altitudes, like those further away from the equator, also experience more temperature variation and environmental harshness [21]. At least in insects, the intensity of predation is also higher at lower altitudes [17].

Like areas at higher latitudes and elevations, islands also have fewer and less diverse predators than comparable areas on the mainland [5]. Reduced predation pressure might explain why animals tend to live in smaller groups on islands [22,23,24]. However, islands also tend to experience milder climates than continental areas at the same latitude with less fluctuation year-round in weather [25]. As per the above argument, increased climate stability could explain the occurrence of smaller groups on islands. Given that islands and their mainland counterparts are more similar in climate near the equator than at higher latitudes [25], the island effect associated with climate might in addition be more pronounced at higher latitudes.

In addition to factors like climate and number of predators, which vary at the biogeographical scale, other smaller-scale ecological factors can potentially shape the evolution of group size. It is important to consider these effects as they might confound the association between group size and ecology at the biogeographical scale. I review briefly ecological factors relevant to pigeons including body size, diet, habitat type, habitat use, and population density. Large species are considered less vulnerable to predation because they are more difficult to subdue [26,27,28]. In addition, large species also have larger eyes, which would increase the ability to detect predators from afar [29]. Therefore, large species should be less likely to live in larger groups. Diet is typically strongly associated with foraging group size. Food items in animal diets broadly occur in clumps or are dispersed spatially [30]. Seeds and fruits are clumped resources typically associated with high within-patch abundance that can accommodate many foragers at the same time. Scattered clumps of seeds or fruits might favour the formation of groups to exploit such resources more efficiently [31]. Diets including nectar or insects, by contrast, inhibit the formation of groups as such resources can be defended or lead to intense competition among group members [11]. As the typical diet in pigeons includes fruits or seeds, variation in diet among species was not expected to play a large role in foraging group size. Habitat type and use can also affect foraging group size. Open habitats with little visual obstacles are thought to increase prey conspicuousness, which should lead to the formation of larger groups to increase the ability to detect predators early [13,32,33,34]. Foraging on the ground among concealing vegetation is thought to reduce predation risk, leading to the formation of smaller groups [34,35]. Finally, low population density might reduce opportunities to join others to form groups and lead to smaller groups [36]. Indeed, empirical studies often show an increase in group size in denser populations [37,38,39].

There have been few attempts to examine how group size varies at broad biogeographical scales [24,40,41,42] and none has simultaneously considered estimates of predation and climatic factors. To fill this gap, I examined how foraging group size varies across a large number of pigeon species as a function of latitude, altitude, and island living taking into account known potential confounding ecological variables. If climatic factors are the primary drivers of foraging group size, I predicted an increase in group size at higher latitudes and altitudes in response to more instability. Smaller groups should also occur on islands and the island effect should become more prominent at higher latitudes. By contrast, if predator richness is the main driver of variation in group size, I predicted larger groups nearer the equator and at low elevations and smaller groups on islands.

I used a phylogenetic framework to control for possible non-independence among closely related species and relied upon published data on foraging group size in pigeons. Pigeon species are found on all continents except Antarctica and often live year-round on islands [43,44]. Pigeon species occur across a broad range of latitudes and altitudes. In addition, pigeons vary in size over two orders of magnitude and can forage alone or in pairs or in groups numbering in the thousands. Pigeon species forage in all types of habitats from open fields to dense forests. Different species forage exclusively on the ground or in the canopy. This broad variation among pigeon species is ideal to examine the drivers of foraging group size at broad biogeographical scales.

## 2. Materials and Methods

### 2.1. Data Collection

Data on foraging group size in pigeons are most frequently reported as a range with a minimum and maximum. The arithmetic mean group size is occasionally reported. However, the use of this metric is not recommended for group size because the occurrence of infrequent large groups alongside the more common smaller groups can overestimate the size of the group typically experienced by individuals [45]. Geometric mean can be used as a central tendency metric for this type of right-skewed data [2]. For each species, geometric mean was obtained by calculating the mean based on the minimum and maximum reported group sizes in log_10_ scale.

I searched the literature for studies reporting foraging group sizes in pigeons worldwide. I started the search with two general monographs on pigeons [43,44] and updated group size data by carrying out searches from primary sources using queries on Google Scholar combining each species name and the keywords flock or group. As I focused on foraging groups, I did not consider group sizes for flying or roosting pigeons if a distinction was made. I also excluded data coming from studies conducted in cities or from species outside their native range to reduce potential anthropogenic impacts on group size. I excluded extinct species or species extinct in the wild.

In addition to minimum and maximum group sizes, I gathered additional information on the ecology of each species. I used published sources to obtain average body mass [46]. I used a published standardized classification for habitat type, diet, and breeding range geographic location and size [47]. This classification distinguished three different types of habitats based on the amount of vegetation present (open, semi-open, closed) and three different types of diets based on the most common items eaten (frugivore, granivore, omnivore). This source also provided the centroid of latitude and longitude for the breeding range of each species and range size (km^2^). Another published standardized classification provided an estimate of habitat use in the form of the percentage of time spent foraging on the ground [48]. To calculate population density for each species, I used mid-range estimates of population size from BirdLife International (https://datazone.birdlife.org/), which I then divided by range size. Species of pigeons were classified as restricted to mountains or lowlands based on estimates of altitude range provided in the above monographs. Species whose altitude range did not include sea level were considered restricted to mountains. I used four variables to quantify climate across the breeding range of each species, namely, annual temperature variation (standard deviation of monthly temperature values over a year), average annual temperature, annual precipitation variation (standard deviation of monthly precipitation values over a year), and average annual precipitation [49]. I did not consider migration as a driver of group size because only a handful of pigeon species are truly migratory. As the range of foraging group sizes is likely to increase with the number of sources used for a given species, research effort was estimated by the number of primary sources used to establish geometric mean foraging group size.

Most species of pigeons occur exclusively on islands or the continent. Some species, however, can occupy both islands and the continent. I classified such species as island living if the breeding range overlapped mostly with islands or vice versa. For less extreme cases, the classification was based on whether the range centroid occurred on an island or the continent.

For each species of pigeons, I determined the number of predator species co-occurring within their breeding range. I only considered avian diurnal birds of prey (Accipitridae and Falconidae) as potential predators because predation on adults is mostly limited to these species [43,44]. I excluded predators considered rare or occasional within the breeding range of a pigeon species or predators with diets restricted to taxa other than birds such as snake- or fish-eaters or scavengers. To determine the number of co-occurring predators, I relied on species lists of birds provided by Avibase for each country in the world along with their administrative divisions (https://avibase.bsc-eoc.org).

For species of pigeons restricted to one island, I counted the number of potential predator species as defined above using the species list for that particular island. For species of pigeons not restricted to one island, I used the species list from the island closest to the range centroid. This is reasonable as individuals in non-endemic species can rarely occupy the full extent of the breeding range and thus typically experience only a subset of the prevailing conditions across their range. By choosing an island close to the range centroid, I selected a region that is most likely typical of what an average individual can experience in terms of co-occurring predators.

The breeding range of continental species rarely matched the boundaries of a single country and often spilled into neighbouring countries. Therefore, I used the species list from the administrative division of a country (e.g., county, province or state) that included or was nearest to the range centroid. The size of administrative divisions is not standardized worldwide. Consequently, the number of predator species present might increase with the size of the administrative division potentially inflating the number of predators occurring in larger divisions. Therefore, I also obtained the area of the administrative division used to establish the number of co-occurring predators for each species.

The number of co-occurring predators is an index of predation pressure that takes into account density (i.e., by excluding rare or occasional predators) and diet (i.e., by excluding predators with non-avian specialized diets). Another factor that might play a role in predation pressure is the size of a pigeon species. Predators tend to avoid prey that are too small or too large for their size. To produce a predator pressure index adjusted for prey body mass, I relied on a published allometric relationship plotting the expected range of prey sizes as a function of predator size [50]. For each species of pigeons, I used the list of potential predator species as determined above and excluded those that were too large or too small to include this particular species of pigeon in their diet. This size-adjusted number was always equal or typically lower than the potential number of predators.

### 2.2. Data Analyses

I used the ‘phylolm’ R-package for the phylogenetic Gaussian linear model [51]. The dependent variable was geometric mean foraging group size for each species (in log_10_ scale). The set of independent variables included: body mass in log_10_ scale, absolute latitude, diet, island living, mountain living, habitat type, habitat use, and research effort. As the effect of island living might vary depending on latitude, I also included the interaction between island living and absolute latitude. Given that population density was missing for many species, I ran a separate model with population density and the above independent variables. Finally, I ran additional models substituting absolute latitude for climatic variables or number of predators while keeping the other ecological variables. Substitution was warranted because latitude probably acts through ecological variables that vary alongside like climatic variables. In models including number of predators, I included size of the administrative division in log_10_ scale as an additional co-factor.

The variance-covariance distance matrix was used to control for phylogenetic relatedness. The matrix was obtained using a consensus tree based of 1000 trees downloaded from the most complete phylogeny of pigeons currently available [52]. Some sub-species of pigeons have been elevated to full species status since this phylogeny was established. To match the phylogeny, I only retained for analysis the sub-species with the most available data for a particular species. The phylogenetic model was run under three different evolutionary scenarios. After comparing AICs for the various models, the best fit was provided by the scenario based on Pagel’s lambda, which was used subsequently for statistical inference. I assessed multicollinearity among the set of independent variables using variance inflation factors (VIF). All VIFs were smaller than 4 showing no multicollinearity issues [53].

## 3. Results

**Figure 1:**
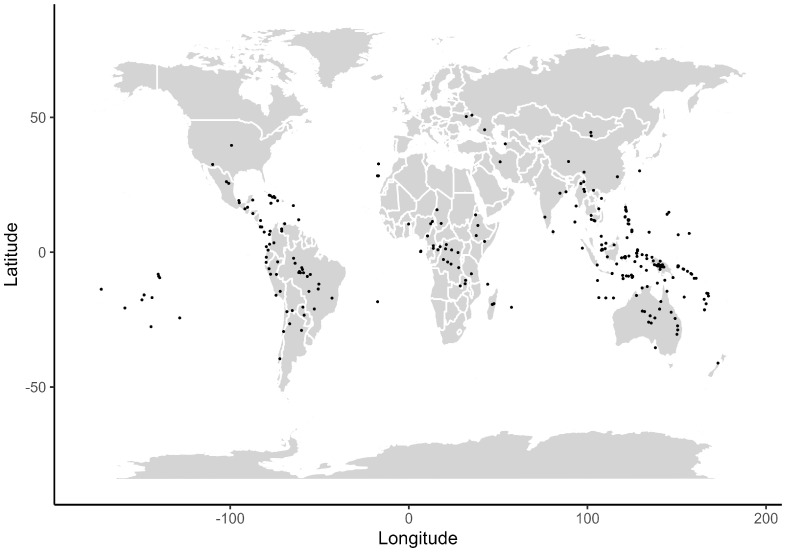
Geographic distribution of pigeon species based on the centroid of latitude and longitude for the breeding range (*n* = 286 species). Each dot represents the location of one species.

I gathered data on foraging group size for 286 pigeon species distributed worldwide (Figure 1). This number represents about 81% of the 353 recognized species in the phylogeny. Much variation occurred among species for the various ecological variables used in the analysis with the following breakdown: location (160 island species, 126 continental species), altitude (61 mountain species, 225 lowland species), diet (100 frugivores, 76 granivores, 110 omnivores), habitat (76 species in closed habitats, 169 in semi-open habitats, 41 in open habitats), habitat use (ranging from 0 to 100% foraging on the ground with 84 species restricted to the ground and 106 species restricted to the canopy). Body mass of the pigeon species ranged across nearly two orders of magnitude (from 28 to 2384 g). Geometric mean foraging group size in pigeon species ranged over three orders of magnitude with genera such as *Leptotila* and *Geotrygon* including many species living alone or in pairs and others such as *Streptopelia* and *Columba* including species living in very large groups with a maximum in the thousands.

The phylogenetic signal in the analysis including absolute latitude was weak (λ = 0). Based on the likelihood R2, the full model explained 46.2% of the variation in foraging group size (Table 1). The full model revealed that geometric mean group size did not vary statistically with body mass or diet. The statistically significant interaction between island living and absolute latitude indicated that geometric mean group size increased at higher latitudes for continental species (β (se) = 0.021 (0.0028), *p* < 0.0001) but not for species living on islands (β (se) = 0.073 (0.075), *p* = 0.33). Geometric mean group size was significantly larger for species restricted to mountains and for species living in more open habitats. In addition, species living closer to the canopy as opposed to the ground lived in statistically larger groups. All these findings were corrected for the positive effect of research effort on geometric mean group size. Population density was available for 103 species. When included in the above model, population density was not associated with geometric mean group size (β (se) = 0.019 (0.034), *p* = 0.58).

**Figure 2:**
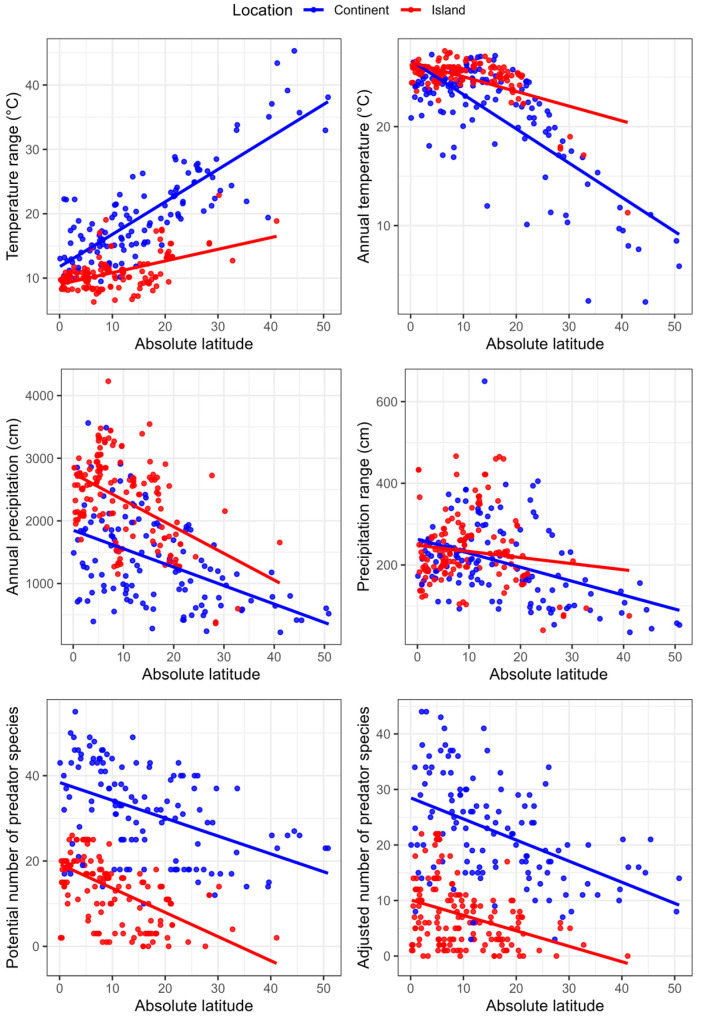
Variation in climatic and predation variables as a function of absolute latitude in species of pigeons living on islands or continents (*n* = 286 species). Linear regression trend lines are shown for descriptive purposes.

Climatic and predator variables all varied with absolute latitude in previously documented patterns (Figure 2). At the descriptive level, annual temperature, annual precipitation, and annual precipitation variation decreased at higher latitudes while annual temperature variation increased. Both the potential number of predators and the size-adjusted number of predators decreased at higher latitudes. For a given absolute latitude, islands were warmer and wetter overall with less annual variation in temperatures. The number of predators was also smaller on islands.

**Figure 3:**
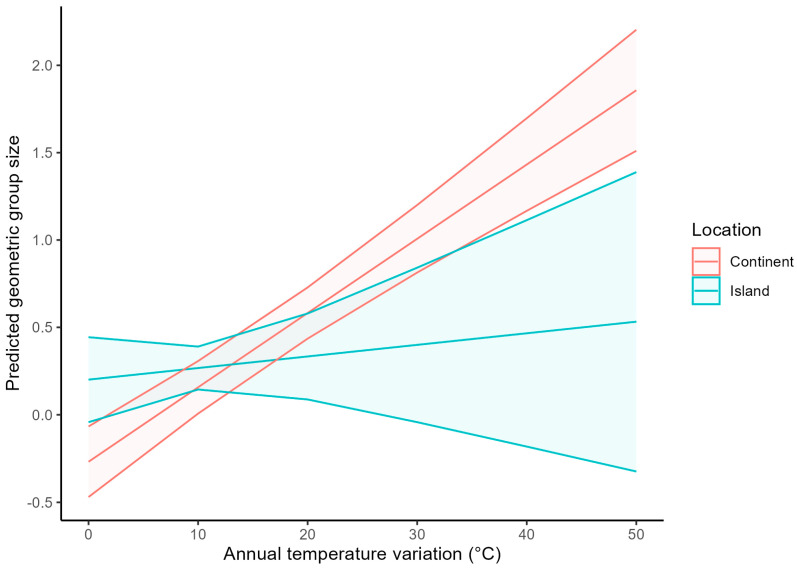
Relationship between predicted geometric mean foraging group size (in log_10_ scale) and annual temperature variation in pigeons living on islands or continents (*n* = 286 species). Linear trend lines and 95% confidence interval ribbons are taken from a phylogenetic model.

Using AICs, I ranked all models substituting absolute latitude by one of the climatic or predator variables while keeping the other ecological variables. The best model included annual temperature variation (AIC = 182.2) distantly followed by the model including annual precipitation (AIC = 222.7), annual temperature (AIC = 232.1), annual precipitation variation (AIC = 244.1), size-adjusted number of predators (AIC = 263.4), and potential number of predators (AIC = 267.2). In the models with predator numbers, there was no significant association between geometric mean group size and the size-adjusted number of predators (β (se) = −0.0049 (0.0034), *p* = 0.15) or the potential number of predators (β (se) = −0.0027 (0.0027), *p* = 0.32). In the model with annual temperature variation, geometric mean group size increased when the annual temperature variation increased for continental species (β (se) = 0.043 (0.0047), *p* < 0.0001) but less so for island species (β (se) = 0.0066 (0.011), *p* = 0.54) (Figure 3).

In a final model, I added the size-adjusted number of predators (with administrative division size as a co-factor) to the model including annual temperature variation and the full set of independent variables. When annual temperature variation was taken into account, the effect of the number of predators remained statistically non-significant (β (se) = 0.0030 (0.0031), *p* = 0.34) while geometric mean group size still increased with annual temperature variation for continental species (β (se) = 0.043 (0.0048), *p* < 0.0001) but not for island species (β (se) = 0.0048 (0.011), *p* = 0.66).

## 4. Discussion

In a phylogenetic analysis of a large, cosmopolitan avian family, I identified several predictors of variation in foraging group size across pigeon species worldwide. Group size varied significantly with latitude, altitude, and island living. Notably, the effect of latitude depended on geographic context: foraging group size increased with latitude among continental species but showed no such trend among island species. Climatic factors and predator richness also varied with latitude. When substituting latitude with these environmental variables, annual temperature variation emerged as the strongest predictor—greater variability was associated with larger groups, but only for continental species. Neither the number nor body-size-adjusted number of predators within a species’ breeding range was significantly related to group size. Additionally, a subset of the data revealed no significant relationship between population density and foraging group size.

The occurrence of a latitudinal gradient in foraging group size has received little attention. An increase in group size with latitude has been reported in parrots [24] but not in tits and chickadees [40]. In contrast, a study on dolphin species found the opposite pattern, with larger groups observed at lower latitudes [41]. Unlike many bird species, dolphins do not typically defend year-round territories, and it has been suggested that the greater food availability in warmer tropical waters may explain this inverse gradient in group size. However, the potential role of predation risk in shaping these patterns remains unexamined in dolphins.

Theories underlying a latitudinal gradient in group size emphasize climate or predation. My findings provide little evidence to support an effect of predation at this scale. The number of predators occurring in the breeding range of a species or the size-adjusted number of predators was poorly associated with group size. There was little evidence that group size was larger at lower latitudes in response to the greater diversity and abundance of avian predators that occur there. In contrast, greater climate stability at lower latitudes predicts smaller group sizes. In addition, islands and continents are expected to diverge more extensively in climate at higher latitudes [25], suggesting that the greatest difference in group size between islands and the continent should occur at higher latitudes, as was the case here. Climate stability was also invoked to explain why clutch size in birds increases at a much faster rate with latitude in continental than in island species [54].

The best predictor of group size in terms of climate was annual temperature variation, lending support to the idea that a more stable climate at lower latitudes, at least in terms of temperature, favours the evolution of smaller group sizes. This is in line with the observation that in birds year-round territoriality is more prevalent in the tropics than in temperate areas [20]. The idea that climate can impact social organization is not new. Climate is thought to play a role in shaping the evolution of other types of social organizations including group living in primates [55] and cooperative breeding in birds and mammals [7,56]. Emerging from these studies, it appears that measures of climate variability rather than averages better predict the occurrence of these societies.

The lack of effect of predation on group size was surprising. While the number of co-occurring predators is admittedly a crude estimate of predation pressure, a similar metric was able to explain other features related to sociality such as the occurrence of family living across many avian families [50] and the size of mixed-species groups in one avian family [40]. My approach to calculate co-occurring predators could be improved by incorporating other considerations such as the spatial overlap in habitat use between predators and prey and the density of predators.

I found that larger groups tended to occur in species living at higher elevations. A similar finding was observed in parrots [24] but not in groups composed of several species of birds [42]. Again here, it appears that predation is not associated with the effect of altitude. The occurrence of fewer predators at higher elevations, reflecting a general reduction in biodiversity as altitude increases [4], would select for smaller rather than larger groups. Higher elevations are associated with more variable climate [21]. As was the case with the latitudinal gradient, this greater variation appears to correlate with larger groups. Key resources at higher altitudes may be less available and more difficult to locate [57] thus favouring the formation of groups. Future studies could document the association between group size and altitude by measuring climatic variables and group size directly in the range of species living at different elevations.

Other ecological variables also explained variation in group size among species of pigeons. Larger groups of birds and mammals tend to occupy more open habitats [13,32,33,34] and this was also the case among pigeons. Greater exposure to predators in more open habitats is thought to drive the need to form large groups to detect predators more efficiently. Similarly, greater protection by surrounding vegetation closer to the ground is thought to reduce the need to form large groups [34,35]. In pigeons, species living closer to the ground were less likely to forage in large groups. This was not the case in parrots [24], but few species of parrots forage exclusively on the ground like in pigeons where several genera such as *Streptopelia*, *Zenaida*, *Columbina*, and *Leptotila* are limited to the ground. Diet is often associated with group size [58,59], but this was not the case in pigeons. Smaller groups tend to occur in species foraging on defensible resources such as nectar or on active prey like insects that can escape when disturbed by nearby companions. The typical diet in pigeons include grains and fruits, which are not impediments to the formation of groups like nectar or insects, perhaps explaining the weak association between diet and group size.

Larger prey species may be more difficult to subdue by predators reducing the need to forage in groups [26,27,28]. This appears not to be the case in pigeons with the largest species such as the crown pigeons (*Goura* spp.) in New Guinea and the imperial pigeons (*Ducula* spp.) often foraging in groups [43]. Across a large number of species from many avian families, body mass was also weakly associated with the likelihood of forming groups [59]. Sparser populations might reduce the ability to form groups [36]. There was little evidence for an association between population density and group size in pigeons. Admittedly, estimates of population density were lacking for many species of pigeons. While more estimates are needed to assess the robustness of the findings, the results thus far suggest that densities of pigeons are generally not a constraint in the ability to form groups.

This study has other limitations. Species of pigeons that have been more extensively studied tend to provide more data on group sizes, which likely results in a broader observed range. To account for this, I controlled for research effort by considering the number of sources used to determine group size for each species. Ideally, research effort should be assessed at the level of individual studies—for example, by recording the number of groups encountered during surveys. However, such detailed information was often unavailable. Furthermore, the use of standardized protocols for estimating group size is essential to minimize methodological inconsistencies across sources [60]. Implementing these improvements would help reduce error in group size estimates.

## 5. Conclusions

The results of this study provide evidence that biogeographical variation in group size in pigeons worldwide is driven by climatic features rather than number of predators. Predation probably plays a more important role at the scale of the habitat.

## Figures and Tables

**Table 1:** Results from a phylogenetic model for the effect of body mass and ecology on foraging group size in pigeons (*n* = 286 species). Estimates are in log_10_ scale. Bold values indicate statistically significant effects.

Variable	Levels	Estimate (SE)	*p*-Value
Intercept		−0.35 (0.19)	0.06
Body mass in log_10_ scale		0.089 (0.073)	0.22
Absolute centroid latitude		0.021 (0.0028)	**<0.0001**
Geographic location	Islands v. continent	0.073 (0.075)	0.33
Interaction between absolute centroid latitude and island living	Islands	−0.017 (0.0046)	**0.0002**
Diet	Granivore v. frugivore	0.10 (0.087)	0.23
	Omnivore v. frugivore	0.0048 (0.077)	0.95
Altitude range	Mountains v. lowlands	0.10 (0.052)	**0.04**
Habitat type	Semi-open v. closed	0.22 (0.056)	**<0.0001**
	Open v. closed	0.32 (0.074)	**<0.0001**
Habitat use (% ground foraging)		−0.0021 (0.00078)	**0.008**
Research effort (number of sources used)		0.55 (0.084)	**<0.0001**

## Data Availability

Data and codes are available from the author.
